# The deep lateral femoral notch sign (DLFNS) is associated with concomitant knee injuries in skeletally immature patients with anterior cruciate ligament injuries

**DOI:** 10.1002/jeo2.70595

**Published:** 2025-12-28

**Authors:** Annika Babette Ito, Maria Christine van der Steen, Robin Nicolaas Julian Voskuilen, Rob Paulus Augustinus Janssen, Martijn Dietvorst

**Affiliations:** ^1^ Department of Orthopaedic Surgery & Trauma Máxima Medical Centre Eindhoven‐Veldhoven the Netherlands; ^2^ Department of Orthopaedic Surgery & Trauma Catharina Hospital Eindhoven the Netherlands; ^3^ Department of Biomedical Engineering Eindhoven University of Technology, Orthopaedic Biomechanics Eindhoven the Netherlands; ^4^ Department of Paramedical Sciences Fontys University of Applied Science Eindhoven the Netherlands

**Keywords:** ACL injury, knee morphology, knee trauma, MRI, paediatric

## Abstract

**Purpose:**

The aim of this study was to investigate the prevalence of the deep lateral femoral notch sign (DLFNS) in skeletally immature patients with anterior cruciate ligament (ACL) injuries and its potential association with trauma‐related and clinical factors.

**Methods:**

A total of 87 skeletally immature patients with ACL injuries and a minimum follow‐up of 1 year were included and matched based on sex and skeletal age with 44 controls. The presence of the DLFNS, defined as a notch depth of >1.5 mm, was assessed on MRI. Intra‐ and inter‐observer reliability were calculated, and prevalence was determined. Associations between the DLFNS and trauma mechanism, trauma‐related bone lesions, concomitant injuries, clinical instability, and treatment at both baseline and follow‐up were assessed using Fisher's exact tests.

**Results:**

The prevalence of DLFNS was 11.5% (10/87) among skeletally immature patients with ACL injuries compared to 0% (0/44) in controls (*p* = 0.02). DLFNS depth demonstrated excellent intra‐ and inter‐observer reliability (ICC > 0.90). Furthermore, a significant positive association was identified between the DLFNS and overall concomitant knee injuries (*p* = 0.02), including ligamentous, meniscus, and cartilage injuries. Further subgroup analysis did not reveal any statistically significant associations with individual injury types. All patients presenting with a DLFNS ultimately received operative treatment, either during initial management or follow‐up. This revealed a significant positive association between the DLFNS and primary operative treatment (*p* = 0.02).

**Conclusion:**

The prevalence of the DLFNS in skeletally immature patients with ACL injuries was 11.5%. When present, the DLFNS was associated with overall concomitant knee injuries and primary ACL reconstruction. The DLFNS may therefore be an important sign to take into consideration on the primary MRI in skeletally immature patients with ACL injuries.

**Level of Evidence:**

Level III.

## Graphical abstract

The deep lateral femoral notch sign (DLFNS) occurs in 11.5% of skeletally immature patients with anterior cruciate ligament injuries and is associated with overall concomitant knee injuries and primary anterior cruciate ligament reconstruction.

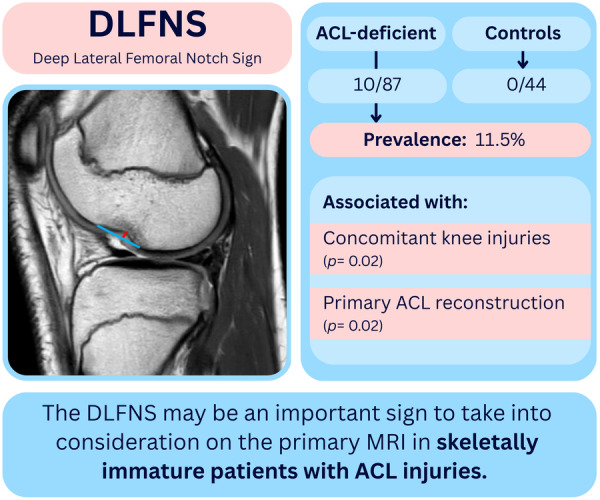

**Abbreviations**
ACL: anterior cruciate ligament
CI: confidence interval
DLFNS: deep lateral femoral notch sign
ICC: intraclass correlation coefficient
IOC: International Olympic Committee
IQR: interquartile range
MRI: magnetic resonance imaging
PAMI: paediatric anterior cruciate ligament initiative
PCL: posterior cruciate ligament
PDW: proton density‐weighted
SD: standard deviation

## INTRODUCTION

The incidence of paediatric anterior cruciate ligament (ACL) injuries has increased over the last decades [12, 24, 34]. In these young active patients, ACL injuries affect quality of life [15] and increase the risk of further knee injuries as well as the development of early‐onset osteoarthritis [1, 33]. Children and adolescents with ACL injuries undergo either non‐operative or operative treatment. In skeletally immature patients, ACL reconstruction is associated with high failure and revision rates, as well as a risk of growth disturbance [5, 21]. Therefore, in line with the International Olympic Committee (IOC) consensus statement, non‐operative treatment is preferred, provided that the patient has no concomitant injuries requiring surgery [1, 9]. A growing body of evidence suggests that delaying surgical treatment until the growth plates have closed is associated with an increased risk of persistent knee instability and meniscal and chondral injuries [20, 21]. This highlights the need for further investigation into various patient characteristics, such as morphological risk factors, that can aid in predicting which skeletally immature patients will benefit from non‐operative treatment and who will require surgery [35]. To support this decision‐making process, Grassi et al. recently developed and validated the ‘BABY‐knee algorithm’ [14]. This algorithm combines clinical and radiological variables to guide treatment in skeletally immature patients with ACL injuries, showing promising results.

Among the factors potentially relevant to these predictive models is a post‐traumatic morphological indicator previously described in association with ACL injuries, the deep lateral femoral notch sign (DLFNS) [11, 17, 26]. During the injury mechanism of ACL injuries, a valgus/internal rotation mechanism results in ventral subluxation of the tibia, causing the lateral femoral condyle to impact the posterolateral tibia plateau [25, 28]. This results in a bony impression on the lateral femoral condyle, commonly referred to as the DLFNS [27]. In skeletally mature patients, the DLFNS was first described on plain lateral radiographs [8] as an indirect sign of ACL injuries and was later identified on magnetic resonance imaging (MRI). Previous studies indicate that the prevalence of the DLFNS in adults with ACL injuries varies from 11.8% to 41.6% based on MRI measurements with a depth cut‐off of >1.5 mm [29]. In addition to indicating ACL injury [11, 26], the DLFNS in skeletally mature patients is associated with lateral meniscal tears [4, 6], medial collateral ligament injuries [13], and rotatory instability [27].

Research on the DLFNS in the paediatric population remains limited. Bernholt et al. reported a prevalence of 36.6% on MRI in ACL‐injured paediatric patients, along with an association between the DLFNS and lateral meniscal tears, as well as medial meniscal ramp lesions [2]. In contrast, Pascual‐Leone et al. reported a lower prevalence of 5.8% on radiographs. They sought to understand the resolution pattern of the DLFNS in paediatric patients, suggesting that the DLFNS has the potential to resolve following ACL reconstruction [31]. However, both studies included children regardless of their physeal status. Therefore, this study aimed to investigate the prevalence of the DLFNS in skeletally immature patients with ACL injuries and its potential association with trauma‐related and clinical factors.

The hypothesis was that the DLFNS would be more prevalent in skeletally immature patients with ACL injuries compared to those without. Additionally, we hypothesised that the DLFNS would be associated with non‐contact trauma, the presence of trauma‐related bone lesions (such as bone bruises and posterolateral tibial impression fractures), the presence of concomitant injuries, increased clinical knee instability, primary operative treatment at baseline, and switch to operative treatment during follow‐up due to non‐coping with non‐operative treatment.

## METHODS

### Patients

This retrospective case‐control study received approval from the local ethics committee of the Máxima Medical Centre (N.23.078 and N.18.089). Consent was obtained from patients or their legal guardians prior to treatment for the scientific use of data obtained as part of standard care. Data were subsequently collected from the patients' electronic medical records.

Patients who met the eligibility criteria were selected from the Dutch contribution to the ESSKA‐Paediatric Monitoring Initiative (PAMI) [30]. Patients were included between October 2018 and May 2025. The ACL‐deficient group was coarsely matched (2:1) with a control group based on sex and skeletal age. The control group was selected from a cohort of patients presenting with knee complaints at the Máxima Medical Centre between 2020 and 2024. The inclusion and exclusion criteria for both groups are described in Table 1.

**Table 1:** Inclusion and exclusion criteria for the ACL‐deficient and control groups.

Criteria	ACL‐deficient	Controls
Inclusion	ACL rupture, diagnosed by MRI and a positive Lachman's test	No intra‐articular pathologies, including but not limited to ACL rupture, ruled out by MRI
	Open growth plates	Open growth plates
	Minimum follow‐up of 1 year after initial diagnostic MRI	
Exclusion	Tibial spine fractures	Concomitant ipsilateral knee injuries (e.g., meniscal, collateral, ACL, and PCL)
	Combined ACL‐PCL injuries	Ipsilateral fractures
	Knee dislocations	Previous ipsilateral surgeries of the knee related to meniscus, ACL, PCL, collateral ligaments, or cartilage injuries
	Insufficient quality of the initial diagnostic MRI: Missing PDW or T1‐weighted sagittal sequences Misaligned MRI images	

Abbreviations: ACL, anterior cruciate ligament; MRI, magnetic resonance imaging; PCL, posterior cruciate ligament; PDW, proton density‐weighted.

### Measurements

#### Patient‐file

Relevant patient characteristics for the ACL‐deficient group, including sex, age at the time of MRI, affected side, hyperlaxity (defined as hyperextension of the contralateral knee of ≥ 10°), and the interval between trauma and MRI, were retrospectively collected from the electronic patient records and analysed. Information on sex, affected side, and age at the time of MRI was also gathered for the control group. Additional parameters of interest that were collected for the ACL‐deficient group included the mechanism of trauma, the presence of concomitant knee injuries on MRI, clinical knee instability assessed through physical examination tests, the choice of treatment at baseline, and the switch to operative treatment during follow‐up. The physical examination tests included the Lachman test, anterior drawer test, and pivot shift test. The outcomes were categorised using the IKDC‐2000 rating system [10, 16].

#### MRI

The primary parameter of interest was the presence of the DLNFS on MRI. Additional performed measurements included skeletal age according to the shorthand MRI Bone Age Assessment Tool of the knee by Politzer et al. [32], the presence of a bone bruise on the lateral femoral condyle and the lateral tibial plateau following the methods outlined by Wang et al. [36], and the presence of a posterolateral tibial plateau impaction fracture classified according to the classification system by Bernholt et al. [2, 3]. Bone bruise measurements were only performed if the initial diagnostic MRI was performed within 8 weeks following trauma [37].

**Figure 1:**
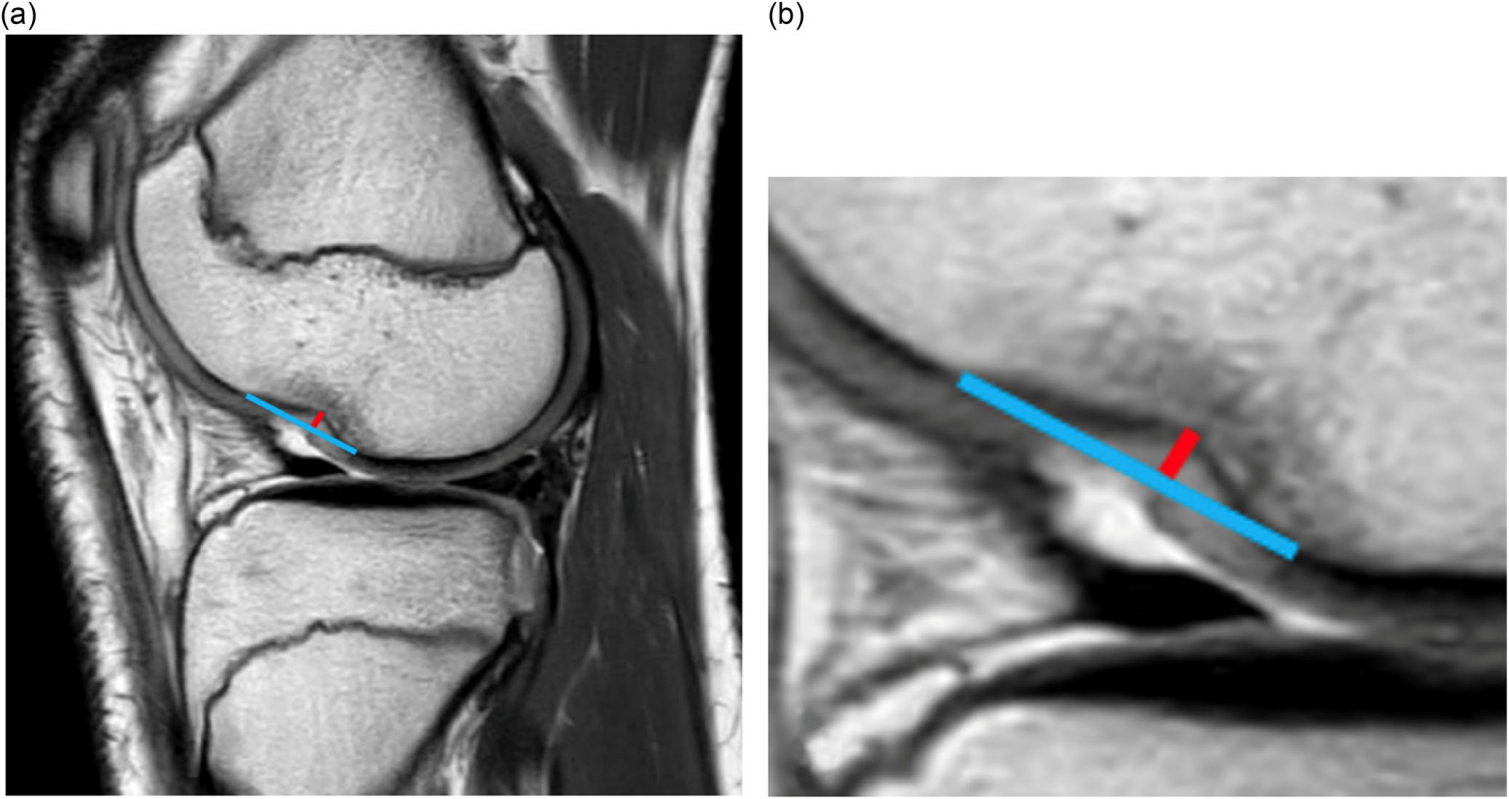
(a) Measurement technique to determine the depth of the deep lateral femoral notch sign (DLFNS) on proton density weighted (PDW)‐ or T1‐weighted sagittal magnetic resonance imaging (MRI). The blue line represents the tangent line connecting the anterior and posterior edges of the impression; the red line indicates the DLFNS depth, defined as the perpendicular distance from the tangent line to the deepest portion of the impression. (b) Zoomed‐in view of (a) to provide a more detailed visualisation of the measurement.

DLFNS depth was measured following the methods outlined by Bernholt et al. (Figure 1) [2, 3, 18]. The presence of an impression on the lateral femoral condyle was identified on the proton density‐weighted (PDW) or T1‐weighted sagittal MRI sequences. The MRI slice with the deepest point of the impression was selected, and a tangent line was drawn, connecting the anterior and posterior edges of the impression. Depth was subsequently measured as the distance between the tangent line and the deepest portion of the impression (Figure 1). A depth > 1.5 mm was considered positive for the presence of a DLFNS [2, 3, 31]. Measurements were conducted by a trained medical student (AI). A randomly selected 25% of the measurements were reassessed after a minimum interval of 2 weeks to determine intra‐observer reliability. To determine inter‐observer reliability, the same measurements were also independently assessed by an orthopaedic resident (RV), who was blinded to the outcome of the measurements conducted by the first observer.

### Statistics

Sample size calculations were conducted using G*Power 3.1 targeting an estimated power of 80% (1‐β) and an alpha level of 0.05. Given the varying prevalence reported in previous research for the DLFNS with a cut‐off point > 1.5 mm, an estimated prevalence of 16.5% was used to determine the sample size [2, 18, 31]. With a 2:1 matched cohort, the required sample size to reach 80% power was 96 (64 cases and 32 controls).

Statistical analyses were performed using IBM SPSS Statistics for Windows (Version 29.0, Armonk, NY, USA). For all analyses, statistical significance was defined as *p* < 0.05. Descriptive statistics were used to describe the study population. The normality of continuous variables was assessed using the Shapiro‐Wilk test. For normally distributed continuous variables, means and standard deviations (SD) were used to express population characteristics. For non‐normally distributed variables, medians, along with the interquartile range (IQR) were reported. Independent t‐tests, chi‐squared tests, Fisher's Exact tests, or Mann‐Whitney tests were used to examine differences in baseline characteristics between patients in the ACL‐deficient and control groups, as well as between the DLFNS‐positive and DLFNS‐negative groups.

The intra‐ and inter‐observer reliabilities of the DLFNS depth measurements were evaluated using a single‐measure Intraclass Correlation Coefficient (ICC) with a two‐way random‐effect model for absolute agreement. An ICC value below 0.5 indicated poor reliability; 0.5–0.75 indicated moderate reliability; 0.75–0.90 indicated good reliability; and values greater than 0.90 were considered indicative of excellent reliability [23].

The prevalence of the DLFNS in the ACL‐deficient group was calculated and compared to the control group using Fisher's Exact test. Additionally, within the ACL‐deficient group, Fisher's Exact tests were conducted to explore the association of the DLFNS with trauma mechanisms, the presence of trauma‐related bone lesions (bone bruises on the lateral femoral condyle or lateral tibial plateau, and posterolateral tibial impression fractures), the presence of concomitant knee injuries, clinical instability, the choice of treatment at baseline, and the switch to operative treatment during follow‐up, both before physeal closure and overall.

## RESULTS

### Patient characteristics

A total of 131 patients met the eligibility criteria and were included in the study, with 87 in the ACL‐deficient group and 44 in the control group. Baseline characteristics are presented in Table 2. Both groups included more males than females. Although no difference was found in skeletal age between the ACL‐deficient group and the control group (*p* = 0.56), calendar age differed significantly (*p* = 0.01).

**Table 2:** Baseline characteristics of the study population.

	ACL‐deficient (*n* = 87) #N (%/IQR)	Controls (*n* = 44) #*N* (%/IQR)	*p* value
Sex			0.56
Female	16 (18.4)	10 (22.7)	
Male	71 (81.6)	34 (77.3)	
Age (years)	14.1 (12.3–15.3)	13.5 (11.4–14.2)	0.01*
Skeletal age			0.56
9 years	0 (0.0)	0 (0.0)	
10 years	1 (1.1)	0 (0.0)	
10.5 years	2 (2.3)	1 (2.3)	
11 years	9 (10.3)	3 (6.8)	
11.5 years	5 (5.7)	2 (4.5)	
12 years	3 (3.4)	1 (2.3)	
13 years	18 (20.7)	12 (27.3)	
14 years	16 (18.4)	9 (20.5)	
15 years	23 (26.4)	10 (22.7)	
16 years	8 (9.2)	1 (2.3)	
17 years	0 (0.0)	0 (0.0)	
<11 years^a^	2 (2.3)	5 (11.4)	
Affected side			0.24
Right	43 (49.4)	17 (38.6)	
Left	44 (50.6)	27 (61.4)	

*Note*: According to the shorthand MRI Bone Age Assessment Tool of the knee by Politzer et al., the lowest available category for boys was 11 years [32]. However, seven boys had a bone age below this threshold and were therefore classified as ‘<11 years’.

Abbreviations: ACL, anterior cruciate ligament; IQR, interquartile range.

* Statistically significant difference (*p* < 0.05).

### DLFNS prevalence

A positive DLFNS ( > 1.5 mm) was identified in 11.5% of patients in the ACL‐deficient group (10/87), while none of the patients in the control group demonstrated a positive DLFNS (0/44) (*p* = 0.02). DLFNS depth measurements demonstrated excellent reliability, with an ICC of 0.997 (95% confidence interval (CI): 0.994–0.999) for intra‐observer agreement and 0.984 (95% CI: 0.966–0.992) for inter‐observer agreement.

### DLFNS in ACL‐deficient patients

The ACL‐deficient group was further divided into two subgroups based on the presence of a DLFNS: the DLFNS‐positive group (*n* = 10) and the DLFNS‐negative group (*n* = 77).

#### Patient characteristics

The baseline characteristics of these groups are presented in Table 3. Patients in the DLFNS‐positive group were older (median = 14.9) than those in the DLFNS‐negative group (median = 13.6) (*p* = 0.04). However, no significant difference was observed in skeletal age (*p* = 0.71). Furthermore, sex, affected side, hyperlaxity, and the time between trauma and MRI in weeks also did not differ significantly between the groups either.

**Table 3:** Baseline characteristics of the DLFNS‐positive and DLFNS‐negative groups.

	ACL‐deficient (*n* = 87)		
	DLFNS‐positive (*n* = 10) #*N* (%/SD/IQR)	DLFNS‐negative (*n* = 77) #*N* (%/SD/IQR)	*p* value
Sex			0.68
Female	1 (10.0)	15 (19.5)	
Male	9 (90.0)	62 (80.5)	
Age (years)	14.9 (±1.2)	13.6 (±1.9)	0.04*
Skeletal age			0.71
9 years	0 (0.0)	0 (0.0)	
10 years	0 (0.0)	1 (1.3)	
10.5 years	0 (0.0)	2 (2.6)	
11 years	0 (0.0)	9 (11.7)	
11.5 years	0 (0.0)	5 (5.6)	
12 years	0 (0.0)	3 (3.9)	
13 years	2 (20.0)	16 (20.8)	
14 years	1 (10.0)	15 (19.5)	
15 years	5 (50.0)	18 (23.4)	
16 years	2 (20.0)	6 (7.8)	
17 years	0 (0.0)	0 (0.0)	
<11 years^a^	0 (0.0)	2 (2.3)	
Affected side			0.31
Right	3 (30.0)	40 (51.9)	
Left	7 (70.0)	37 (48.1)	
Hyperlaxity b	0 (0.0)	5 (6.8)	1.00
Time between trauma and MRI (weeks)	14.4 (4.0–20.6)	9.1 (3.9–24.1)	0.72

Abbreviations: ACL, anterior cruciate ligament; DLFNS, deep lateral femoral notch sign; IQR, Interquartile range; SD, standard deviation.

^a^ According to the shorthand MRI Bone Age Assessment Tool of the knee by Politzer et al., the lowest available category for boys was 11 years [32]. However, seven boys had a bone age below this threshold and were therefore classified as '<11 years'.

^b^ Data was missing for 5 patients (1 in the DLFNS‐positive group and 4 in the DLFNS‐negative group).

* Statistically significant difference (*p* < 0.05).

#### Parameters of interest

The relationships between the DLFNS and the parameters of interest are shown in Table 4. There was no association between the DLFNS and mechanisms of trauma or trauma‐related bone lesions. However, a significant positive association was found between the DLFNS and the presence of overall concomitant injuries (*p* = 0.02). Further subdivision revealed a trend toward a higher incidence of meniscal injuries in the DLFNS‐positive group. Regarding clinical instability, no association was observed across any of the physical examination tests.

**Table 4:** Relationships between the DLFNS and parameters of interest.

	DLFNS‐positive (*n* = 10) #*N* (%)	DLFNS‐negative (*n* = 77) #*N* (%)	*p* value
Trauma mechanism^a^			1.00
Non‐contact	8 (80.0)	55 (73.3)	
Contact	2 (20.0)	20 (26.7)	
Trauma‐related bone lesions			
Bone bruise lateral femoral condyle^b^	3 (100.0)	21 (63.6)	0.54
Bone bruise later tibial plateau^b^	2 (66.7)	18 (54.5)	1.00
Posterolateral tibial impaction fracture	2 (20.0)	4 (5.2)	0.14
Concomitant injuries	8 (80.0)	30 (39.0)	0.02*
Ligamentous	2 (20.0)	9 (11.7)	0.61
Medial meniscus	3 (30.0)	11 (14.3)	0.20
Lateral meniscus	3 (30.0)	14 (18.2)	0.40
Cartilage	1 (10.0)	3 (3.9)	0.39
Clinical instability^c^			
Lachman^d^			0.61
0–2 mm	0 (0.0)	0 (0.0)	
3–5 mm	0 (0.0)	2 (2.8)	
6–10 mm	5 (50.0)	45 (63.4)	
>10 mm	5 (50.0)	24 (33.8)	
Anterior drawer test^e^			1.00
0–2 mm	0 (0.0)	0 (0.0)	
3–5 mm	0 (0.0)	6 (8.6)	
6–10 mm	9 (90.0)	58 (82.9)	
>10 mm	1 (10.0)	6 (8.6)	
Pivot shift test^f^			0.33
Normal	0 (0.0)	1 (1.5)	
Glide (+)	2 (20.0)	7 (10.4)	
Clunck (+)	7 (70.0)	53 (79.1)	
Gross (+++)	1 (10.0)	1 (1.5)	
Not possible	0 (0.0)	5 (7.5)	
Treatment			
Baseline			
Primary operative treatment	5 (50.0)	12 (15.6)	0.02*
Follow‐up^g^			
Switch to operative treatment	5 (100.0)	38 (61.3)	0.15
Switch to operative treatment prior to physeal closure	1 (20.0)	11 (17.7)	1.00

Abbreviations: DLFNS, deep lateral femoral notch sign.

^a^ Data was missing for 2 patients in the DLFNS‐negative group.

^b^ Bone bruise assessment was not performed in 51 patients (7 in the DLFNS‐positive group and 44 in the DLFNS‐negative group), due to MRI scans obtained more than 8 weeks post‐trauma (*n* = 46) or missing sagittal T2‐weighted sequences (*n* = 5).

^c^ 5 patients from the DLFNS‐negative group were excluded.

^d^ Data was missing for 1 patient in the DLFNS‐negative group.

^e^ Data was missing for 2 patients in the DLFNS‐negative group.

^f^ Data was missing for 5 patients in the DLFNS‐negative group.

^g^ A total of 70 patients who received non‐operative treatment were included in the follow‐up analysis: 5 in the DLFNS‐positive group and 65 in the DLFNS‐negative group. Subsequently, 3 patients from the DLFNS‐negative group were excluded due to loss to follow‐up.

* Statistically significant difference (*p* < 0.05).

All patients presenting with a DLFNS ultimately received operative treatment for their ACL injury, either during initial management or follow‐up. At baseline, patients in the DLFNS‐positive group underwent primary operative treatment more frequently than those in the DLFNS‐negative group, revealing a significant positive association between the DLFNS and primary operative treatment (*p* = 0.02). The remaining patients received primary non‐operative treatment (*n* = 70) and were included for follow‐up analysis. However, three patients were lost to follow‐up and subsequently excluded, resulting in a DLFNS‐positive group of five patients and a DLFNS‐negative group of 62 patients. No associations were found between the DLFNS and the switch to operative treatment during follow‐up, including switch to operative treatment prior to physeal closure.

## DISCUSSION

The most important finding of this study was the significant positive association between the DLFNS and the presence of overall concomitant injuries and primary operative treatment in skeletally immature patients with ACL injuries. The prevalence of the DLFNS was 11.5% (10/87) in skeletally immature patients with ACL injuries, compared to 0% (0/44) in the controls. These findings suggest that the DLFNS may be an important additional sign to consider when assessing knee trauma and injury in this population.

Previous studies on the DLFNS have reported varying prevalence rates. Mostowy et al. conducted a scoping review on the DLFNS in the adult ACL‐injured population, reporting a prevalence ranging from 11.8% to 46.1% when a cutoff value of >1.5 mm was used to identify the DLFNS on MRI [29]. To the best of current knowledge, only two prior studies have examined the prevalence of the DLFNS in the paediatric population. Pascual‐Leone et al. reported a prevalence of 5.8% based on radiographs using the same cut‐off value of > 1.5 mm. This prevalence is lower than that previously reported in the adult population. Therefore, the authors suggested that the DLFNS might be less common or shallower in paediatric patients [31]. In contrast, Bernholt et al. reported a prevalence of 36.3% on MRI when using the same cut‐off value [2]. While both studies focused on the paediatric population, children were included regardless of their physeal status. Bernholt et al. did, however, report a separate prevalence of 32.1% based on the included patients with open growth plates. The higher prevalences reported by Bernholt et al. may be attributed to their broader definition of a positive DLFNS, which included not only the cut‐off value of >1.5 mm but also a second area of concavity distinct from the sulcus terminalis [2]. This broader definition of a positive DLFNS is not commonly used in previous literature and may therefore have led to higher prevalence outcomes. In the present study, focus was solely on children with open growth plates, providing important context to the existing literature on DLFNS prevalence in the skeletally immature population. It indicates that while the DLFNS might not be as prevalent as in adults, it still occurs notably among children with ACL injuries.

Additionally, this study found a significant positive association between the DLFNS and the presence of overall concomitant injuries in skeletally immature patients with ACL injuries. This aligns with the literature presented by Mostowy et al. in their scoping review on the DLFNS in the adult population [29]. Previous research has focused more often on the relationship between the DLFNS and meniscal injuries, particularly of the lateral meniscus. In the present study, although not statistically significant, a higher incidence of meniscal injuries was observed in patients with a DLFNS compared to those without (60.0%, 6/10 vs. 31.2%, 24/77). This lack of significance may be attributed to the small number of patients with DLFNS, limiting subgroup analysis. Similarly, Bernholt et al. assessed the relationship between DLFNS and meniscal injuries in paediatric patients, finding a significant positive association specifically between the DLFNS and lateral meniscus tears and medial meniscus ramp lesions [2]. These findings align with recent studies conducted in adults, showing that lateral meniscal injuries are more common in patients with ACL injuries who present with a DLFNS than in those without a DLFNS [6, 25]. The relationship between the DLFNS and lateral meniscal injuries is thought to result from the trauma mechanism that causes the DLFNS, where the lateral femoral condyle impacts the posterolateral corner of the tibial plateau [4, 6].

Regarding clinical instability, the present study did not find an association with the DLFNS in skeletally immature patients with ACL injuries. There is no prior literature on this topic within the paediatric population. Previous research in adults has examined this relationship with mixed results. Chang et al. found no significant association between the DLFNS ( > 1.5 mm) and the degree of knee joint laxity in both acute ( < 3 months) and chronic ( > 3 months) patients with ACL injuries. Knee joint laxity was assessed using Lachman and pivot shift tests performed the day before ACL reconstruction [7]. Similarly, Huang et al. demonstrated that the presence of the DLFNS ( > 2.0 mm) did not indicate greater dynamic tibial laxity measured using an arthrometer 6 months after injury [19]. Kanamamedala et al. assessed instability under general anaesthesia using a validated tablet‐based image analysis software and a triaxial accelerometer during a standardised pivot shift test. They did not find any correlation between the DLFNS (> 2.0 mm) and quantitative measures of rotatory instability [22]. In contrast, Lucidi et al. did report a correlation between the DLFNS (> 2.0 mm) and increased rotatory laxity. They assessed rotatory laxity intraoperatively through a kinematic analysis of the pivot shift test using a surgical navigation system. However, when comparing patients with a DLFNS between 1 and 2 mm to those without a DLFNS, they found no significant difference in rotatory laxity [27]. These mixed results may be attributed to the varying methods used to assess instability, as well as the different time points at which the assessments were conducted. Since the current study relied on standard care data from electronic medical records, clinical stability was evaluated using reported outcomes of the Lachman‐, anterior drawer, and pivot shift tests. These tests were executed by experienced orthopaedic surgeons during the first consultation following trauma, to establish a consistent comparison across patients. Long‐term studies following the development of instability over time may help identify which children are at risk of developing symptomatic clinical instability. This could provide a better understanding of the potential relationship between the DLFNS and clinical instability.

In the present study, all skeletally immature patients presenting with a DLFNS ultimately underwent operative treatment for their ACL injury, either during initial management or follow‐up. At baseline, 50% (5/10) of patients with a DLFNS underwent primary ACL reconstruction, compared to only 15.6% (12/77) of those without a DLFNS. Interestingly, this demonstrated a significant positive association between the DLFNS and primary operative treatment. In the Netherlands, the current standard of care for skeletally immature patients focuses on primary non‐operative treatment, in line with the recommendations of the 2018 IOC consensus statement [1, 9]. A lower number of skeletally immature patients undergoing primary ACL reconstruction was therefore expected. According to this consensus statement [1], primary non‐operative treatment is a safe and viable option for skeletally immature patients without associated injuries or major instability issues. However, primary ACL reconstruction is recommended for skeletally immature patients with repairable concomitant injuries requiring surgery or those facing unacceptable participation restrictions. The high percentages of patients with a DLFNS undergoing primary operative treatment may be related to the previously described association between the DLFNS and overall concomitant injuries, which serve as indicators for primary ACL reconstruction. This supports the idea that the DLFNS may serve as an additional factor for assessing knee trauma and injury, highlighting its potential role in supporting treatment decision‐making.

The present study had several limitations. Since data collection for secondary outcomes relied on existing electronic medical records, a risk of information bias exists. However, this risk was minimised by reducing variability in clinical assessment, as only two experienced orthopaedic surgeons conducted consultations and documentation following a predefined outline. Another limitation was the range of follow‐up period among patients, which meant that not all patients were monitored until physeal closure. As a result, some patients may still switch to operative treatment in the future. This introduces a potential risk for censoring bias and may influence the results. However, all patients in the current study who presented with a DLFNS either received operative treatment at baseline or had already switched to operative treatment during the follow‐up of this study. Therefore, since all data for this subgroup were available, the influence on the results was minimal. Lastly, although the required sample size for determining prevalence was achieved, the small number of patients with a DLFNS may have limited the identification of potential relationships within this subgroup. Therefore, larger studies are needed to confirm the findings and to explore possible additional associations. An international multi‐centre database is essential to provide robust data for future research [1]. The Paediatric Anterior Cruciate Ligament Initiative (PAMI), launched by the European Society for Sports Traumatology, Knee Surgery & Arthroscopy, offers such a database [30]. However, at present, data on the DLFNS are not yet recorded for all patients included.

## CONCLUSION

The prevalence of the DLFNS in skeletally immature patients with ALC injuries was 11.5%. When present, the DLFNS was associated with overall concomitant knee injuries and primary ACL reconstruction. The DLFNS may therefore be an important sign to take into consideration on the primary MRI in skeletally immature patients with ACL injuries.

## AUTHOR CONTRIBUTIONS

All authors contributed to the conception and design of the study. Annika Babette Ito and Robin Nicolaas Julian Voskuilen participated in data acquisition. Annika Babette Ito analysed the data and drafted the manuscript. Annika Babette Ito, Maria Christine van der Steen, Martijn Dietvorst, and Rob Paulus Augustinus Janssen were involved in data interpretation. Annika Babette Ito, Maria Christine van der Steen, Robin Nicolaas Julian Voskuilen, Rob Paulus Augustinus Janssen, and Martijn Dietvorst critically revised the manuscript. Maria Christine van der Steen supervised the research project. All authors have read and approved the final version of the manuscript and agree to be accountable for all aspects of the work, ensuring that any questions related to the accuracy or integrity of any part are appropriately investigated and resolved.

## CONFLICT OF INTEREST STATEMENT

The authors declare no conflicts of interest.

## ETHICS STATEMENT

The Medical Ethical Committee at the Máxima Medical Centre determined that the rules laid down in the Medical Research Involving Human Subjects Act (MWO) do not apply to this study. The local ethics committee of the Máxima Medical Centre approved the conduct of this study (Reference numbers: N.23.078 and N.18.089). Informed consent was obtained from all patients or their legal guardians prior to treatment for the scientific use of clinical data as part of standard care.

## Data Availability

The data supporting the findings of this study are available upon request from the corresponding author (AI). The data are not publicly available due to privacy or ethical restrictions.
